# Comparison of Machine Learning Models for Classification of Breast Cancer Risk Based on Clinical Data

**DOI:** 10.1002/cnr2.70175

**Published:** 2025-04-02

**Authors:** Haniyeh Rafiepoor, Alireza Ghorbankhanloo, Kazem Zendehdel, Zahra Zangeneh Madar, Sepideh Hajivalizadeh, Zeinab Hasani, Ali Sarmadi, Behzad Amanpour‐Gharaei, Mohammad Amin Barati, Mozafar Saadat, Seyed‐Ali Sadegh‐Zadeh, Saeid Amanpour

**Affiliations:** ^1^ Cancer Biology Research Center Cancer Institute, Tehran University of Medical Sciences Tehran Iran; ^2^ School of Industrial Engineering, Iran University of Science and Technology Tehran Iran; ^3^ Department of Industrial Engineering Iran University of Science and Technology Tehran Iran; ^4^ Osteoporosis Research Center, Endocrinology and Metabolism Research Institute Tehran University of Medical Sciences Tehran Iran; ^5^ School of Medicine, Tehran University of Medical Science Tehran Iran; ^6^ Faculty of Mechanical Engineering, K. N. Toosi University of Technology Tehran Iran; ^7^ School of Mechanical Engineering, University of Tehran Tehran Iran; ^8^ Department of Mechanical Engineering School of Engineering, University of Birmingham Birmingham UK; ^9^ Department of Computing School of Digital, Technologies and Arts, Staffordshire University Stoke‐on‐Trent UK

**Keywords:** artificial intelligence, breast cancer, conventional models, machine learning, risk assessment

## Abstract

**Background:**

Breast cancer (BC) is a major global health concern with rising incidence and mortality rates in many developing countries. Effective BC risk assessment models are crucial for prevention and early detection. While the Gail model, a traditional logistic regression‐based model, has been broadly used, its predictive performance may be limited by its linear assumptions. With the rapid advancement of artificial intelligence (AI) in medical sciences, various complex machine learning algorithms have been developed for risk prediction, including for BC.

**Aims:**

This study aims to compare the quality of AI‐based models with the traditional Gail model in assessing BC risk using a population dataset. It also evaluates the performance of these models in predicting BC risk.

**Methods and Results:**

This study involved 942 newly diagnosed BC patients and 975 healthy controls at the Cancer Institute in IKH hospital Complex, Tehran. Ten classification algorithms were applied to the dataset. The accuracy, sensitivity, precision, and feature importance in the machine learning algorithms were assessed and compared to previous studies for evaluation. The study found that AI algorithms alone did not significantly improve predictability compared to the Gail model. However, the importance of variables varied significantly among the AI algorithms. Understanding feature importance and interactions is crucial in AI modeling in order to enhance accuracy and identify critical risk factors.

**Conclusion:**

This study concluded that, in BC risk prediction, incorporating specific risk factors, such as genetic and image‐related variables, may be necessary to further enhance accuracy in BC risk prediction models. Furthermore, it is crucial to address modeling issues in models with a restricted number of features for future research.

**Abbreviations**
BC: breast cancer
BCRAT: breast cancer risk assessment tool
DT: decision tree
IARC: International Agency for Research on Cancer
KNN: *k*‐nearest neighbor
LR: logistic regression
ML: machine learning
RF: random forest
SVM: support vector machine

## 1 Introduction

Estimates from the International Agency for Research on Cancer (IARC) in GLOBOCAN 2020 suggest that breast cancer (BC) is the most common cancer globally, with over 2 million new cases annually [1]. In developing countries such as Iran, the incidence and mortality of BC are increasing. Iran reported approximately 17 000 new cases and over 4800 deaths in GLOBOCAN 2020, showing an increase from 13 000 new cases in 2018 [2]. Given the high prevalence and the burden on healthcare systems, the importance of BC prevention has become even more significant. The Gail model, a well‐known BC risk assessment tool (BCRAT), is a comprehensive logistic regression‐based model developed in 1989 to assess BC risk in over 28 000 American women [3]. Based on this model, six factors—age, number of breast biopsies, age at first birth, number of first‐degree relatives with BC, race/ethnicity, and age at menarche—influence the risk of BC.

Logistic regression is a classification model that utilizes maximum likelihood functions to estimate the probabilities of various outcomes. It is traditionally employed to analyze right‐censored data [4]. The primary advantages of logistic regressions are their clarity, interpretability, and lack of assumptions about the distribution of the explanatory data [5]. However, logistic regressions are constrained by their lack of statistical complexity, as they presuppose a linear relationship between the input variables and the log odds of the outcome [6]. Over the past few years, due to the rapid advancement of artificial intelligence (AI) in medical sciences, various complex modern algorithms, including different machine learning (ML) and deep learning methods, have been developed for breast cancer risk prediction [7, 8, 9, 10]. Despite previous efforts to construct BC prediction models using ML algorithms [7, 8, 11, 12, 13, 14], there are currently limitations in the predictive performance of traditional and ML‐based risk prediction models. This study is aimed at evaluating the accuracy of 10 ML‐based models compared to traditional models for predicting BC using parameters from the Gail model. It used the Iranian population dataset from the Rostami et al. study [15].

## 2 Material and Methods

### 2.1 Study Design

This study used data from a hospital‐based, case–control investigation conducted at the Cancer Institute in IKH hospital Complex, Tehran, from September 23, 2011, to May 16, 2016. The recruitment of cases and controls and the study design were detailed in a previous publication [16]. In total, there were 942 newly diagnosed patients with In situ or invasive BC as incident cases. The 975 healthy controls were frequency‐matched to the cases by 5‐year age categories and residential locations. Participants in both the case and control groups were interviewed utilizing a structured questionnaire designed to gather comprehensive data on various sociodemographic characteristics, anthropometric measurements, menstrual and reproductive history, age at menarche, parity, family history of BC, age at first pregnancy, and duration of breastfeeding. These interviews were conducted at the hospital by trained interviewers who were unaware of the study's hypotheses. Interviewers visited the surgery and chemotherapy wards to identify patients who were admitted for treatment at the Cancer Institute. Patients were explained the study and asked to sign a written informed consent form before participating in the interview. Patients' personal information was numerically coded to protect their privacy. Eligible patients included those with a histopathologically confirmed diagnosis of either In situ or invasive BC, who were at least 18 years of age, had no history of concurrent cancer in other organs, and had been newly diagnosed with cancer within the 12 months preceding the interview. Patient recruitment was limited to those who were hospitalized for treatment in the surgery and oncology wards and occurred 3 days a week due to logistic issues. For each case, a control individual was chosen among healthy female acquaintances of patients admitted to Imam Khomeini Hospital Complex for non‐cancer‐related illnesses. The controls were selected to be frequency‐matched by age (within 5‐year intervals), place of residence (Tehran or other provinces), and recruited around the same period as the cases. The control group was evaluated based on the absence of any BC diagnosis or related conditions in the preceding 12 months. BC patients were prompted to disclose their exposure status during the year leading up to their diagnosis. These controls were not associated with the cancer patients. Out of the 1324 eligible controls invited, 967 (73%) participated in the study, while 357 (27%) declined to participate. The details of the study design and establishment of this case–control have been previously described in Maleki et al. [15, 16]. This study was approved by the National Research Ethics Committee, Ministry of Health and Medical Education (code number: IR.TUMS.IKHC.REC.1399.454, Date: December 2020), and the authors accessed the data on January 15, 2021.

### 2.2 Data Pre‐Processing

Gail variables needed to be extracted from the dataset. They were used for training and validating the algorithms. The dataset was divided into 80% for training and 20% for validation.

### 2.3 Model Development

In this study, a total of 10 classification algorithms were systematically applied to a dataset aimed at BC risk assessment. The algorithms utilized in this analysis included decision tree (DT), bagging decision tree (Bagging‐DT), random forest (RF), logistic regression (LR), support vector machine (SVM), bagging support vector machine (Bagging‐SVM), gradient boosting, AdaBoost, XGBoost, *k*‐nearest neighbor (KNN), and statistically inspired modification of partial least squares (SMPLS). The evaluation process was structured around both training and validation datasets, with the validation cohort consisting of 384 subjects. This subset was generated through an automated random sampling technique, representing 20% of the total population of 1917 participants. The prediction models were constructed using all variables. To quantify the performance and reliability of each classification model, several statistical metrics were calculated, including accuracy, precision, and sensitivity. These metrics are critical for assessing the effectiveness of predictive models and were derived through cross‐validation (CV) methodologies. Specifically, the leave‐one‐out CV procedure was employed in the SIMPLS analysis to derive *Q* ^2^ (goodness of prediction) and *R* ^2^ *Y* (goodness of variation) values. The optimal prediction model was determined based on the maximum values of accuracy and *Q* ^2^, ensuring that these metrics did not show a decline, which would indicate potential overfitting. Furthermore, it was essential that *R* ^2^ *Y* exceeded *Q* ^2^, as this relationship serves as a safeguard against overfitting, thereby enhancing the model's generalizability. Grid search was used to fine‐tune hyperparameters for all methods. In this study, the grid search hyperparameter tuning algorithm was employed for several key reasons: (1) Exhaustiveness: Grid search examines every possible combination of hyperparameters, ensuring the identification of an optimal solution. (2) Simplicity and clarity: The grid search method is straightforward and easy to implement. As a comprehensive exploratory algorithm, grid search evaluates the performance of hyperparameters across all potential configurations. It systematically tests each unique combination within the search space to identify the one that yields the best performance [17]. In the context of DTs, several key hyperparameters are critical for model performance that were used in this study. These include the maximum tree depth (*max_depth*), the minimum number of samples required to split an internal node (*min_sample_split*), and the minimum number of samples necessary to be at a leaf node (*min_samples_leaf*). In the KNN model, the hyperparameter *k* (the number of nearest neighbors) is utilized for optimization through grid search. Grid search also optimizes the parameters of SVM, specifically *C*, *γ*, and degree using a CV technique as a performance metric to identify optimal hyperparameters. This study primarily focuses on two parameters of the RF classifier. The grid search incorporates the maximum tree depth, the minimum number of samples, *max_features* (which denotes the maximum number of variables used in individual trees), and *n_estimators* (which indicates the total number of trees to be constructed in the forest). In the gradient boosting method, *max_depth*, *min_sample_split*, and min*_samples_leaf* are considered as hyperparameters that are tuned using grid search. AdaBoost can sometimes be challenging to tune due to its numerous hyperparameters. In this instance, we will perform grid search on two key hyperparameters for AdaBoost: the number of trees used in the ensemble and the learning rate. We will employ a range of well‐performing values for each hyperparameter. Additionally, we will define a grid of hyperparameters, including *max_depth*, learning*_rate*, and *n_estimators* in the XGBoost model, and subsequently conduct grid search.

The algorithms, training, and validation processes were all developed and implemented using the Python programming language. version 3.8.3 and Scikit‐learn library version 0.23.2 and the class GridSearchCV available in Scikit Learn is used for this study.

### 2.4 DT

DTs are a popular supervised learning algorithm used for both classification and regression tasks. They work by recursively partitioning the input space based on the feature that provides the maximum information gain at each step. This results in a tree‐like structure where internal nodes represent decision rules and leaf nodes represent the final predictions. DTs are known for their interpretability, ability to handle both numerical and categorical data, and robustness to outliers. However, they can be prone to overfitting, especially on complex datasets [18].

### 2.5 Bagging‐DT

Bagging, short for Bootstrap Aggregating, is an ensemble learning technique that can be applied to DTs to improve their stability and accuracy. In Bagging‐DT, multiple DT models are trained on random subsets of the training data, and their predictions are combined through majority voting (for classification) or averaging (for regression) to make the final prediction. This helps reduce the variance of the individual DTs and improve the overall model performance [19].

### 2.6 RF

RF is another ensemble learning method that is built on the concept of DTs. It creates a collection of DTs, each trained on a random subset of the features. The final prediction is made by aggregating the predictions of the individual trees. This approach helps to reduce the overfitting problem associated with individual DTs and thus improves the model's generalization ability. RF is widely used for both classification and regression tasks and is known for its robustness to noise and outliers [20].

### 2.7 LR

LR is a supervised learning algorithm primarily used for binary classification problems. It models the probability of a binary outcome as a function of the input features by using a logistic sigmoid function. LR is simple to implement, interpretable, and performs well on linearly separable datasets. Nevertheless, it may struggle with non‐linear relationships and high‐dimensional data [21].

### 2.8 SVM

SVMs are a class of supervised learning algorithms that can be used for both classification and regression tasks. SVMs work by finding the optimal hyperplane that separates the classes with the maximum margin. They are particularly effective in high‐dimensional feature spaces and can handle non‐linear relationships using kernel functions. SVMs are known for their strong generalization performance, but they can be sensitive to the choice of hyperparameters [22].

### 2.9 Bagging‐SVM

Similar to Bagging‐DT, Bagging can also be applied to SVMs to create an ensemble model called Bagging‐SVM. In this approach, multiple SVM models are trained on random subsets of the training data, and their predictions are combined to make the final prediction. Bagging‐SVM can improve the stability and accuracy of individual SVM models, especially on complex or noisy datasets [23].

### 2.10 Gradient Boosting

Gradient Boosting is an ensemble learning technique that combines multiple weak learners, often DTs, to eventually create a strong predictive model. It works by iteratively adding new models to the ensemble, where each new model is trained to correct the errors made by the previous models. Gradient Boosting is known for its high performance on a wide range of tasks and its ability to handle various types of data [24].

### 2.11 AdaBoost

AdaBoost, short for Adaptive Boosting, is another ensemble learning algorithm that combines multiple weak learners, typically decision stumps, to create a strong classifier. It works by iteratively adjusting the weights of the training examples, focusing more on the misclassified instances in each iteration. AdaBoost is known for its ability to improve the performance of weak learners and its robustness to overfitting [25] and is well described in the term of cancer prediction in the study of Kumar et al. [26].

### 2.12 XGBoost

XGBoost, or Extreme Gradient Boosting, is a highly efficient and scalable implementation of the Gradient Boosting algorithm. It incorporates several optimizations, such as regularization, parallel processing, and efficient handling of sparse data, making it a powerful tool for a wide range of ML tasks, including classification, regression, and ranking [27].

### 2.13 KNN

KNN is a non‐parametric, instance‐based learning algorithm used for both classification and regression problems. It works by finding the K closest training examples to a new input and using their labels or values to make a prediction. KNN is simple to implement, can handle non‐linear relationships, and is robust to noisy data. However, it can be computationally expensive for large datasets and may suffer from the curse of dimensionality [28].

### 2.14 Partial Least Squares

A method for partial least squares (PLS) regression, known as the SIMPLS, computes the PLS factors by directly combining the original variables in a linear manner. The PLS factors are chosen to optimize a covariance criterion while adhering to specific constraints related to orthogonality and normalization [29, 30].

### 2.15 Partition Analysis

To classify the continuous and ordinal data values effectively, a partition analysis was conducted by employing a DT algorithm. This analysis aimed to partition the data set in a way that would identify the optimal cutoff points for variables, taking into consideration the relationship between the outcome and the predictors. By utilizing the DT method, the study sought to determine the most appropriate segmentation of the data that would enhance the understanding of the predictive power of the variables in relation to the outcome variable.

### 2.16 Assessing the Importance of Features in ML Algorithms

The effect of each feature on the results was investigated by deleting that feature from the input and checking the result changes while the data was shuffled for each investigation. All the possible permutations of the features were tested using accuracy changes as the measure.

## 3 Results

### 3.1 Patients' Characteristic

All participants were selected from a hospital‐based, case–control study conducted in 2016 at the Cancer Institute of Iran, Tehran. A total of 1917 women, including 942 cases and 975 controls, were chosen to participate. The gathered data contained registry data plus Gail model variables (Table 1).

**TABLE 1:** Patients' characteristics.

Variables	Code	Numbers		Total mean	
		Cases (*n* = 942)	Controls (*n* = 975)	Cases (*n* = 942)	Controls (*n* = 975)
Age at diagnosis (AGECAT)					
< 50	0	579 (61.5%)	638 (65.4%)	47.19 (±10.93)	44.99 (±10.99)
> 50	1	363 (38.5%)	337 (34.6%)		
Age of menarche (AGEMEN)					
≥ 14	0	431 (45.8%)	438 (44.9%)	13.29 (±1.69)	13.41 (±1.66)
12–13	1	377 (40%)	435 (44.6%)		
< 12	2	134 (14.2%)	102 (10.2%)		
Number of biopsies (NBIOPS)					
0	0	869 (92.2%)	960 (98.5%)	0.95 (±0.45)	0.02 (±0.15)
1	1	67 (7.1%)	13 (1.3%)		
≥ 2	2	6 (0.6%)	2 (0.2%)		
Age at first live birth (AGEFLB)					
< 20 or null parity	0	424 (45%)	573 (58.8%)	22.39 (±5.36)	20.02 (±4.58)
20–24	1	281 (29.8%)	284 (29.1%)		
25–29	2	149 (15.8%)	78 (8%)		
≥ 30	3	88 (9.3%)	40 (4.1%)		
Number of first‐degree relatives with breast cancer (NUMREL)				0.09 (±0.36)	0.03 (±0.19)
0	0	870 (92.4%)	947 (97.1%)		
1	1	58 (6.2%)	25 (2.6%)		
≥ 2	2	14 (1.5%)	3 (0.3%)		

### 3.2 Models' Characteristics

**FIGURE 1:**
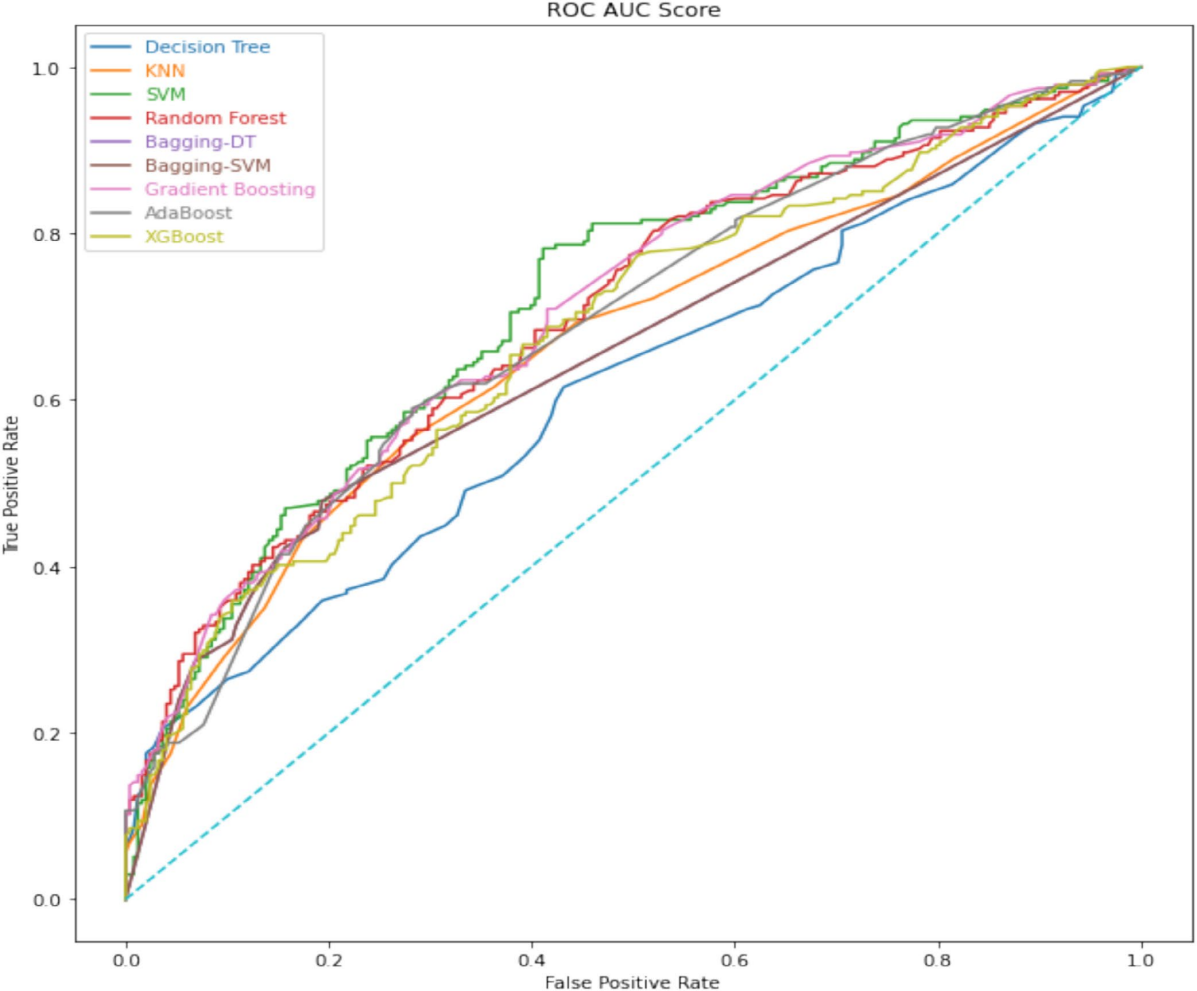
ROC curves for all algorithms on the validation set. The highest validation accuracy was related to gradient boosting (AUC = 0.65).

The outcomes derived from the application of 10 ML algorithms are outlined in Table 2. Within the context of this study, the focal variable pertained to the patient's condition post 5 years subsequent to the prognostication of BC risk from the date of the interview. Table 2 exhibits four key metrics—namely, training accuracy, validation accuracy, sensitivity, and precision—for the various ML techniques employed. Notably, the training datasets revealed that the highest accuracies achieved were 70.88% for the Bagging‐DT and 70.08% for the DT model. In contrast, the validation datasets indicated that AdaBoost attained the highest accuracy at 64.73%, followed closely by Gradient Boosting at 64.52%. Additionally, both Bagging‐DT and Bagging‐SVM exhibited the highest sensitivity rates, recorded at 55.13%. The SVM model demonstrated the greatest precision, achieving a rate of 74.58%. To visually illustrate the predictive performance of these ML methods on the validation set, Receiver Operating Characteristic (ROC) curves were generated and are presented in Figure 1. Furthermore, the SIMPLS model was assessed using quality metrics, yielding a *Q* ^2^ value of 0.084 and an *R* ^2^ *Y* value of 0.068, which serve as indicators of model quality.

**TABLE 2:** Performance comparison of various classifiers. Gradient Boosting emerges as the most accurate ML algorithm.

Approach	Train accuracy	Validation accuracy	Sensitivity	Precision
Decision tree	0.700801	0.574689	0.478632	0.574359
KNN	0.659840	0.636929	0.491453	0.672515
SVM	0.645592	0.634855	0.376068	0.745763
Random forest	0.695459	0.632780	0.482906	0.668639
Bagging‐DT	0.708816	0.628631	0.551282	0.635468
Bagging‐SVM	0.642030	0.628631	0.551282	0.635468
Gradient boosting	0.666963	0.645228	0.517094	0.675978
AdaBoost	0.647373	0.647303	0.538462	0.670213
XGBoost	0.706144	0.616183	0.470085	0.643275
Rostami et al.	0.63		—	—

### 3.3 The Importance of ML Algorithm Features

In Table 3, we present the crucial variables identified by different ML algorithms, along with their relative ranks in descending order. Among the five ML algorithms, namely DT, SVM, RF, Bagging‐SVM, and Gradient Boosting, the variable age at first live birth (AGEFLB) emerged as the top‐ranked risk factor. This indicates that AGEFLB had the highest importance in predicting the patient's condition post 5 years subsequent to the prediction of BC risk. Additionally, AGEFLB secured the second rank in three other algorithms, SIMPLS, Bagging‐DT, and AdaBoost, further highlighting its significance. However, it is worth noting that age at diagnosis (AGECAT) and number of biopsies (NBIOPSIS) exhibited variations in their rankings across the different ML models. This suggests that these variables had differing levels of importance in predicting the patient's condition depending on the specific ML algorithm employed. The variability in rankings underscores the complexity of the predictive models and the diverse ways in which different algorithms weigh the importance of risk factors. This result gave insights into the relative importance of variables across various ML algorithms, enabling them to understand which factors play a significant role in predicting BC risk and subsequent patient outcomes.

**TABLE 3:** Top five key risk factors in descending order for various ML algorithms.

	Decision tree	SVM	Random forest	Bagging‐DT	Bagging‐SVM	Gradient boosting	AdaBoost	XGBoost	SIMPLS	Rostami et al.
1	AGEFLB	AGEFLB	AGEFLB	AGECAT	AGEFLB	AGEFLB	AGECAT	AGECAT	NBIOPS	NBIOPS
2	AGECAT	NBIOPS	AGECAT	AGEFLB	NBIOPS	NBIOPS	AGEFLB	AGEMEN	AGECAT	NUMREL
3	NBIOPS	NUMREL	NBIOPS	AGEMEN	NUMREL	NUMREL	AGEMEN	AGEFLB	NUMREL	AGEFLB
4	AGEMEN	AGEMEN	AGEMEN	NBIOPS	AGEMEN	AGEMEN	NUMREL	NUMREL	AGEMEN	AGECAT
5	NUMREL	AGECAT	NUMREL	NUMREL	AGECAT	AGECAT	NBIOPS	NBIOPS	AGEFLB	AGEMEN

*Note:* AGEFLB and AGECAT are considered the top two predictors among ML algorithms.

Abbreviations: AGECAT: age at diagnosis; AGEFLB: age at first live birth; AGEMEN: age of menarche; NBIOPS: number of biopsies; NUMREL: number of first‐degree relatives with breast cancer.

### 3.4 Predictive Partition Analysis

**FIGURE 2:**
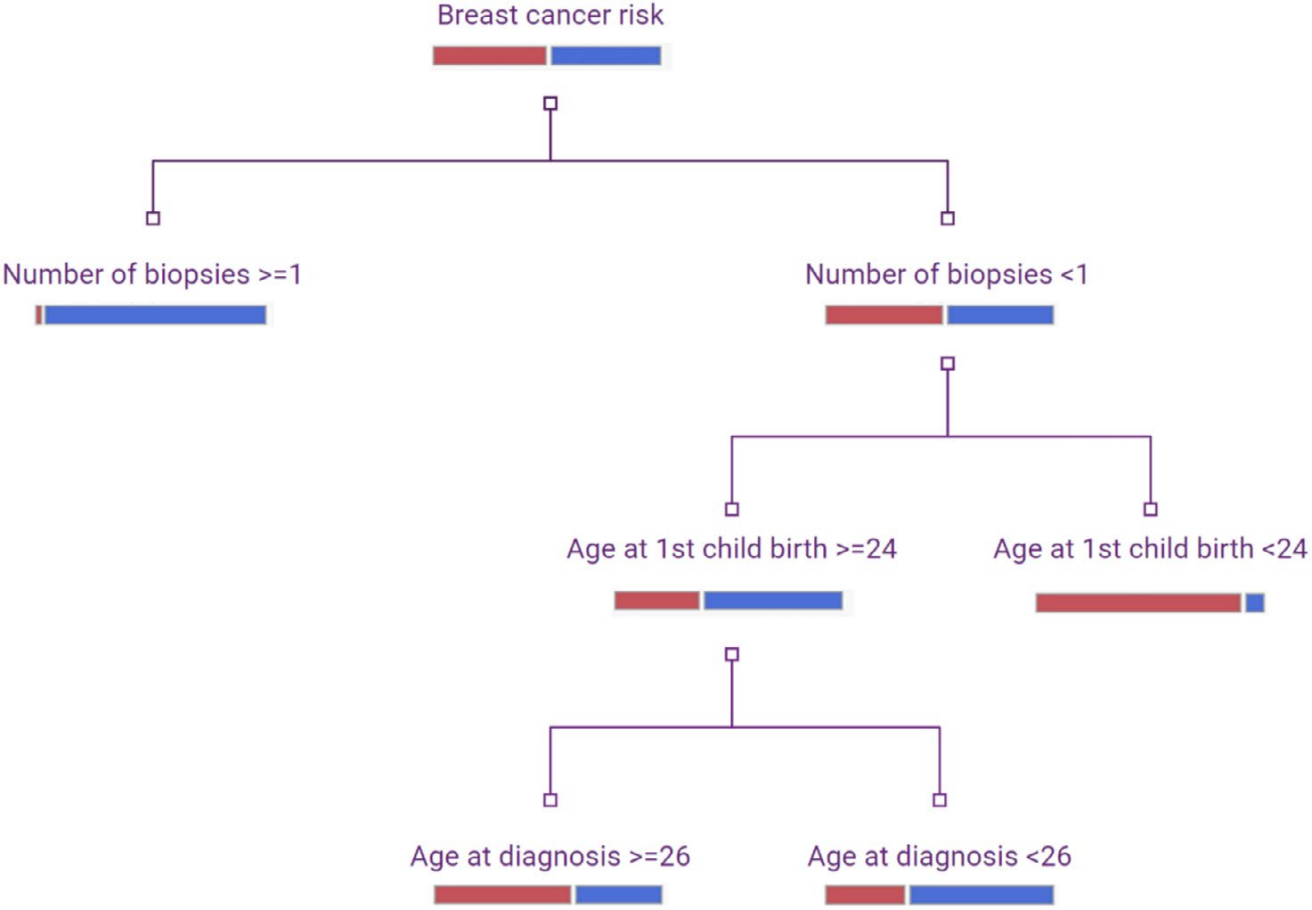
Prediction partition analysis of breast cancer risk prediction. Red: Cases, blue: Controls.

The analysis of BC risk prediction, using partition analysis, revealed two primary branches on either side. These branches indicated that patients with a history of one or more biopsies, an age at first childbirth of less than 24, and an AGECAT of less than 26 were linked to the prediction of BC risk, and this combination of factors could predict the risk with an accuracy of 64%. This finding suggests that further evaluation with a larger population is necessary to assess the identification of cut points for risk prediction (Figure 2).

## 4 Discussion

We examined if ML algorithms can enhance the accuracy of the Gail BC prediction model in Rostami et al.'s study [15]. In this process, we analyzed 10 ML‐based algorithms that included Gail model indicators (age, NBIOPSIS, age at first birth, number of first‐degree relatives with BC, and age at menarche) to assess their predictive accuracy and quality features like sensitivity and precision. The average accuracy of ML‐based models was determined to be 0.63, a result consistent with the findings of Rostami et al., indicating that the AI algorithms alone did not significantly improve the predictability of the model. The SIMPLS model with *Q* ^2^ = 0.0.084 also shows the same result. However, there were notable differences in the importance of variables between the ML algorithms and traditional models. The model development process likely involved various ML‐based analytical approaches, including hyperparameter optimization methods. Comprehending the importance and interactions of features is crucial in AI modeling [31]. Giving a higher weight to a specific feature can significantly improve model accuracy. Conversely, omitting that feature can lead to a notable decrease in accuracy and, thus, diminish the model's utility [32]. In this study, the importance ranking of variables varied significantly across different algorithms, with some assigning a high rank to a feature while others assigned it a low rank (Table 3). However, the final accuracy of the models was generally consistent. Consequently, considering the relationships and correlations between features in AI modeling can greatly facilitate the identification of crucial risk factors and enhance model accuracy with better comprehension of the modeling process.

BC risk prediction models are typically categorized into two types based on the statistical analysis used: traditional and AI models. Traditional models can be categorized into three groups based on their prediction outcomes. The first group includes models that predict the risk of BC, stratified by the risk factors utilized in model development, such as demographic and hereditary factors. The second group comprises models that predict the risk of genetic mutations inheritance, while the third group incorporates both [33]. AI models are divided into two major groups, employing genetic and demographic risk factors for model design. However, certain AI models incorporate histopathological and radiological images through deep learning and convolutional neuronal network (CNN) analysis [34]. In recent years, there has been a rise in the extensive utilization of AI models, aimed at increasing the accuracy of the models. Nevertheless, divergent outcomes have been reported in various researches in terms of BC risk prediction [35]. Therefore, there is a probability that the observed increase in accuracy of the models is not due to the type of algorithms used, but rather it is related to the features being used. For instance, models considering indicators such as genetic factors, radiological images, and other strongly correlated risk factors of BC showed higher accuracy in comparison with similar models. In a systematic review conducted by Gao et al. [36], it has been shown that using image features and genetic risk factors are able to increase the area under curve (AUC) from 0.61 to 0.73 and 0.71 to 0.76 respectively in ML‐based BC risk prediction models. This difference is also presented in the studies of Louro et al. [8] and Cintolo‐Gonzalez et al. [34], which examined and compared traditional models. On the other hand, the observed high accuracy noted in models such as Ming et al. [9] (AUC = 0.91) and Rajendran et al. [37] (AUC = 0.98) used “Personal history of cancer” and “Previous breast procedures” as risk factors, respectively, which strongly correlated with breast neoplasms. As expected, the incorporation of a greater number of intricate features tends to enhance the accuracy of predictive models. However, this augmentation also results in increased model complexity, potentially hindering the feasibility of utilizing the models for practical applications, such as patient assessment via online services. Consequently, this trade‐off may ultimately yield models with reduced accuracy but with simpler user interfaces. This study is subject to some limitations, the consideration of patient demographics in the clinical assessment of an individual's risk may reduce the generalizability of the study findings [38]. Also, this publication lacks longitudinal follow‐up data for the healthy controls. In conclusion, by applying and comparing both ML‐based and traditional models, their AUC ranges were close. According to various research, it has been suggested that enhancing the accuracy of the model requires adding special risk factors such as genetic and image‐related variables. Altering the algorithms alone does not appear to be adequate for increasing the accuracy of a breast risk assessment model. The lack of a special advantage of AI‐based models for predicting the risk of BC in comparison with traditional models, observed in this study, highlights some limitations inherent in the AI modeling process, particularly in models that run with a limited number of features.

## Author Contributions

Conceptualization: Haniyeh Rafiepoor, Alireza Ghorbankhanloo, Kazem Zendehdel, Saeid Amanpour. Data curation: Haniyeh Rafiepoor, Sepideh Hajivalizadeh, Zeinab Hasani. Formal analysis: Zahra Zangeneh Madar, Ali Sarmadi, Behzad Amanpour‐Gharaei, Mohammad Amin Barati, Seyed‐Ali Sadegh‐Zadeh. Funding acquisition: Mozafar Saadat, Seyed‐Ali Sadegh‐Zadeh, Saeid Amanpour. Methodology: Kazem Zendehdel. Supervision: Seyed‐Ali Sadegh‐Zadeh, Saeid Amanpour, Kazem Zendehdel. Visualization: Sepideh Hajivalizadeh, Behzad Amanpour‐Gharaei. Writing – original draft: Haniyeh Rafiepoor, Alireza Ghorbankhanloo. Writing – review and editing: Haniyeh Rafiepoor, Alireza Ghorbankhanloo, Seyed‐Ali Sadegh‐Zadeh, Kazem Zendehdel, Saeid Amanpour.

## Ethics Statement

This study was approved by the ethics committee of IKHC–Tehran University of Medical Sciences, Tehran, Iran (No. IR.TUMS.IKHC.REC.1399.454).

## Conflicts of Interest

The authors declare no conflicts of interest.

## Data Availability

The data that support the findings of this study are available on request from the corresponding author. The data are not publicly available due to privacy or ethical restrictions.
